# Role of transcription factor acetylation in the regulation of metabolic homeostasis

**DOI:** 10.1007/s13238-015-0204-y

**Published:** 2015-09-03

**Authors:** Joo-Man Park, Seong-Ho Jo, Mi-Young Kim, Tae-Hyun Kim, Yong-Ho Ahn

**Affiliations:** Department of Biochemistry and Molecular Biology, Yonsei University College of Medicine, 50-1 Yonsei-ro, Seodaemun-gu, Seoul 120-752 Republic of Korea; Brain Korea 21 PLUS Project for Medical Sciences, Yonsei University College of Medicine, 50-1 Yonsei-ro, Seodaemun-gu, Seoul 120-752 Republic of Korea

**Keywords:** metabolic homeostasis, transcription factor, post-translational modification, type 2 diabetes mellitus

## Abstract

Post-translational modifications (PTMs) of transcription factors play a crucial role in regulating metabolic homeostasis. These modifications include phosphorylation, methylation, acetylation, ubiquitination, SUMOylation, and O-GlcNAcylation. Recent studies have shed light on the importance of lysine acetylation at nonhistone proteins including transcription factors. Acetylation of transcription factors affects subcellular distribution, DNA affinity, stability, transcriptional activity, and current investigations are aiming to further expand our understanding of the role of lysine acetylation of transcription factors. In this review, we summarize recent studies that provide new insights into the role of protein lysine-acetylation in the transcriptional regulation of metabolic homeostasis.

## INTRODUCTION

Chronic metabolic disorder has become a worldwide health problem, in parallel with the increasing incidence of obesity and type 2 diabetes mellitus (T2DM) (Chen et al., 2012). Metabolic syndrome is a disorder caused by abnormal energy utilization and storage (Zivkovic et al., 2007). The pathogenesis of metabolic syndrome involves both insulin resistance and β-cell dysfunction (Kahn et al., 2006). In the insulin-resistance state, the peripheral tissues do not respond to normal circulating concentrations of insulin due to abnormalities in metabolic control mechanisms (Perry et al., 2014). Understanding molecular mechanism(s) of PTM may provide a novel background for develop drugs for anti-metabolic syndrome.

In the adaptation of eukaryotic cells to internal and external stimuli, transcription factors act as critical mediators coordinately regulating biological processes (Francis et al., 2003). In most cases, these transcription factors exert their actions by directly binding to cognate consensus sequences of target genes (Li et al., 2015). These transcription factors are subjected to post-translational modifications (PTMs) affecting their activity, stability, intracellular distribution, and interaction with other proteins (Nerlov, 2008). Types of PTMs include reversible acetylation, phosphorylation, SUMOylation, glycosylation, and ubiquitination (Meek and Anderson, 2009; Zhao et al., 2011). Recently, acetylation and deacetylation of histones and nonhistone proteins have been shown to be involved in the control of cellular energy metabolism (Kim et al., 2006; Guan and Xiong, 2011).

Protein acetylation on lysine residues is regulated by two types of enzymes, histone acetyltransferases (HATs) and histone deacetylases (HDACs). HATs transfer acetyl groups to lysine residues of the substrate proteins, while HDACs catalyze the reverse reaction (Haigis and Sinclair, 2010; Choudhary et al., 2014). HAT/HDAC families are responsible for the fine control of energy metabolism by regulating the transactivation of transcription factors.

Although many recent reviews have described the acetylation and deacetylation of nonhistone proteins, including transcription factors (Glozak et al., 2005; Wang et al., 2011; Choudhary et al., 2014), specific acetylation of the transcription factors associated with regulation of metabolic genes and its relevance to metabolic homeostasis have not been well described. In this review, we will limit our discussion to the relevance of acetylation of transcription factors (excluding nuclear receptors) involved in glucose and lipid metabolism.

## HATS AND HDACS

### Introduction to HATs and HDACs

**Figure 1:**
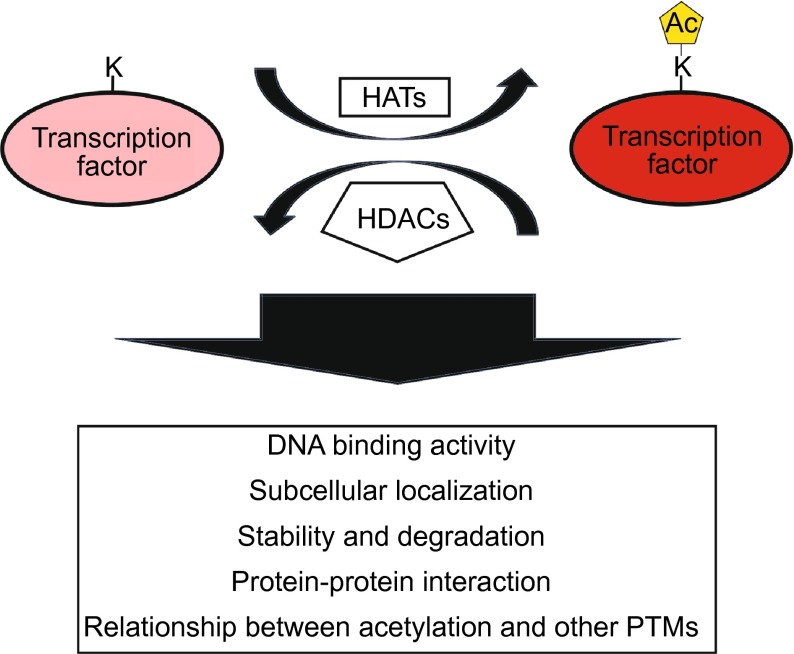
**Functional consequences of acetylation of transcription factors.** Ac, acetyl group; K, lysine residue

In recent decades, acetylation of histones and nonhistone protein has been shown to be a ubiquitous phenomenon that occurs in eukaryotic cells (Vahid et al., 2015). The opposing activities of HATs and HDACs can modulate the activities of diverse transcription factors and regulatory proteins (Glozak et al., 2005; Wang, 2011). The effects of acetylation on various transcription factors are illustrated in Fig. 1. Historically, HATs have been divided into two types depending upon their cellular localization. Type A HATs acetylate both histones and nonhistone proteins in the nucleus. Type B HATs, on the other hand, catalyze the acetylation of histones in the cytoplasm (Lee and Workman, 2007). Recently, some HATs have been shown to function within both the nucleus and cytoplasm and have even been shown to act in the form of multiple complexes (Kimura et al., 2005). Thus, the previously supported classification of HATs into these two groups has become outdated (Carrozza et al., 2003; Allis et al., 2007). At present, there are three major families of HATs: Gcn5-related N-acetyltransferases (GNATs), MYST proteins, and cAMP-response element-binding protein (CBP)/E1A-associated protein of 300 kDa (p300) (Lee and Workman, 2007).

Acetylated proteins are deacetylated by HDACs. There are three classes of mammalian HDACs, which are classified based on their homology to their yeast counterparts. Recently, a fourth subfamily of HDACs was added based on phylogenetic analysis (Shirakawa et al., 2013). Class I HDACs constitute HDAC1, -2, -3, and -8 and are primarily localized to the nucleus. Class II HDACs include HDAC4, -5, -6, -7, -9, and -10 and shuttle between nucleus and cytoplasm (Khan and La Thangue, 2012). Class III HDACs, termed sirtuins, act as NAD+-dependent deacetylases or ADP-ribosyltransferase (Imai and Guarente, 2014). There are seven sirtuins (SIRT1-7), which are distributed in the nucleus, cytoplasm, and/or mitochondria (Chang and Guarente, 2014; Imai and Guarente, 2014).

### Physiological role of HATs and HDACs in the regulation of metabolic processes

Knockout of HATs/HDACs in mice generally results in embryonic lethality (Lagger et al., 2002; Rebel et al., 2002; Gorrini et al., 2007; Montgomery et al., 2008; Gabay et al., 2013). Consequently, studies on the physiological effects of HATs/HDACs require the creation of conditional Cre/LoxP-knockout alleles and heterozygous mice. Among HATs, CBP/p300 is a well-known player involved in regulation of glucose and lipid metabolism. The phenotypes of heterozygous CBP-deficient mice show increased insulin sensitivity and glucose tolerance despite prominent lipodystrophy of white adipose tissue (Yamauchi et al., 2002). Expression of a CH1 domain deletion mutant of CBP/p300 results in improvement of insulin sensitivity with reduction in white adipose tissue and body mass (Bedford et al., 2011). Additionally, disruption of CBP/p300 using adenoviral short hairpin RNA (shRNA) leads to decreased hepatic glucose production (HGP), whereas overexpression of p300 impairs glucose homeostasis and insulin sensitivity (Bricambert et al., 2010; He et al., 2013). Mice with a glycine-to-serine mutation (G422S) of p300 exhibit decreased HGP, suggesting that phosphorylation at serine residues within p300 cause decreases in acetyltransferase activity (He et al., 2012; He et al., 2013). In the HDAC family, HDAC3 and SIRT1 have been well studied in association with metabolic homeostasis. SIRT1 heterozygous knockout mice fed a high-fat diet (HFD) show increased expression of lipogenic genes, and the mice exhibit increased risk of fatty liver development (Xu et al., 2010). Moreover, liver or adipose tissue-specific SIRT1-knockout mice exhibit increased body weight with development of metabolic dysfunction when fed an HFD (Purushotham et al., 2009; Chalkiadaki and Guarente, 2012). Conversely, the gain of SIRT1 function improves glucose homeostasis and insulin-sensitivity in diabetic mice models (Banks et al., 2008; Li et al., 2011). In contrast, liver-specific HDAC3-knockout mice show significant lipid accumulation resulting from increased triglycerides (TG) and overexpression of peroxisome proliferator-activated receptor gamma (PPARγ) genes (Knutson et al., 2008). However, these mice show improved insulin sensitivity and lowered HGP when excess pyruvate and lactate are used for synthesis of TG (Sun et al., 2012). Also, suppression of class IIa HDACs (HDAC4, -5, and -7) ameliorates glucose homeostasis in mice with diet-induced obesity (DIO) mice due to inhibition of hepatic gluconeogenesis (Mihaylova et al., 2011). The physiological roles of HATs/HDACs are summarized in Table 1.

**Table 1:** Summary of the physiological roles of HATs and HDACs

Acetylase/Deacetylase	Loss/Gain of function	Physiological roles	References
CBP	Loss	Increased insulin sensitivity and glucose tolerance	Yamauchi et al. (2002)
CBP/p300	Loss	Improved insulin sensitivity with reduction in body mass Decreased glucose production	Bedford et al. (2011) He et al. (2012)
p300	Gain	Impaired insulin sensitivity and glucose tolerance	Bricambert et al. (2010)
SIRT1	Loss	Impaired insulin sensitivity and glucose tolerance Increased fatty liver development	Chalkiadaki and Guarente (2012) Xu et al. (2010)
	Gain	Improved insulin sensitivity and glucose tolerance	Banks et al. (2008), Li et al. (2011)
HDAC3	Loss	Improved insulin sensitivity and glucose tolerance, despite of increased lipid accumulation	Sun et al. (2012)
Class IIa HDAC	Loss	Improved glucose tolerance by inhibiting gluconeogenesis	Mihaylova et al. (2011)

## ROLE OF TRANSCRIPTION FACTOR ACETYLATION IN THE REGULATION OF GLUCOSE AND LIPID METABOLISM

### Forkhead box O (FoxO) family proteins

**Figure 2:**
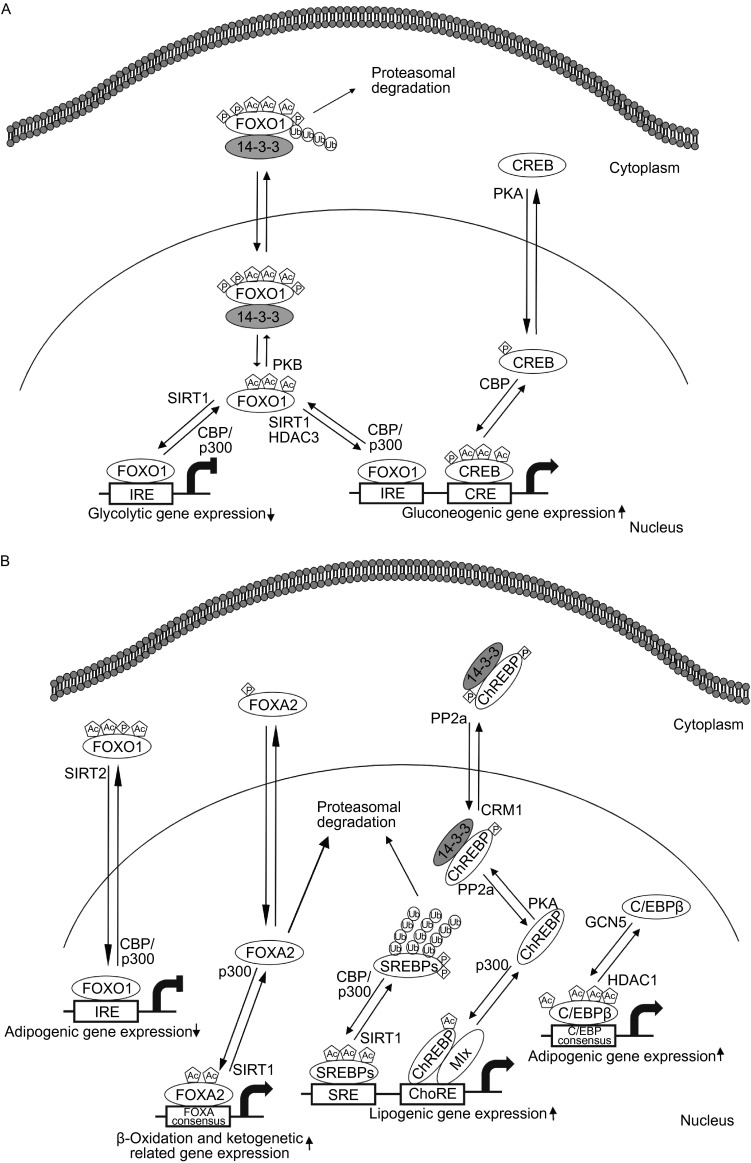
**Role of transcription factor acetylation on the regulation of glucose**. (A) Effect of FOXO1 and CREB acetylation on the carbohydrate metabolism. Acetylation of FOXO1 is balanced by SIRT1 and CBP/p300. Once acetylated, 14-3-3 binds to FOXO1, and localizes to cytosol. The 14-3-3-FOXO1 complex is subjected to proteosomal degradation in cytosol. Deacetylated form of FOXO1 binds to IRE and transactivates glycolytic genes or gluconeogenic genes in a negative or a positive way, respectively. Acetylated form of CREB transactivates gluconeogenic gene expression by binding to CRE in the gluconeogenic genes. Acetylation of CREB is promoted when CREB is phosphorylated. (B) Effect of transcription factor acetylation on the expression of lipogenic genes. Deacetylated form of FOXO1 downregulates adipogenic gene expression by binding to IRE of respective genes. FOXA2 is acetylated by p300 and upregulates expression of genes involved in β-oxidation and ketogenesis. Deacetylated form of FOXA2 is subjected to proteosomal degradation. SREBP family is also acetylated by CBP/p300. Acetylated form of SREBPs upregulates lipogeneic gene expression. Deacetylated form of SREBPs undergo proteasomal degradation. Acetylated form of ChREBP by p300 upregulates gene expression of lipogenic genes. Deacetylated form of ChREBP is phosphorylated by PKA, which promotes binding of 14-3-3, resulting in cytosolic localization. Acetylation of C/EBPβ is balanced by GCN5 and HDAC1. Acetyl-C/EBPβ upregulates adipogenic gene expression. Ac, acetyl group; P, phosphorylation; Ub, ubiquitination; IRE, insulin-response element; CRE, cAMP-response element; PKA, protein kinase A; SRE, SREBP response element; PP2a, protein phosphatase 2a; CRM1, chromosome region maintenance 1 protein

Forkhead box O (FoxO) family proteins including FoxO1, -3a, -4, and -6 are a subfamily of the forkhead group of transcription factors and play a pivotal role in energy metabolism in peripheral tissues (Eijkelenboom and Burgering, 2013). In particular, FOXO1 (also known as FKHR) is important for regulating genes of glucose and lipid metabolism (Matsumoto et al., 2007; Nakae et al., 2008). Phosphorylation of FOXO1 causes cytoplasmic localization resulting in inhibition of its transcriptional activities (Fig. 2) (Maiese et al., 2008). Recently, the transactivation of FOXO1 was shown to be regulated in response to growth factors, starvation, hypoxic stress, and oxidative stress (van der Horst and Burgering, 2007; Calnan and Brunet, 2008). Acetylation of FOXO1 occurs at three lysine residues (Lys242, Lys245, and Lys262) and is facilitated by HATs, such as CBP and p300 (Table 2) (Matsuzaki et al., 2005; van der Heide and Smidt, 2005). Interestingly, the consequences of acetylation of FOXO1 are similar to those of phosphorylation; acetylation of FOXO1 by CBP/p300 leads to reduced DNA-binding activity and translocation to the cytoplasm (Fig. 2) (Matsuzaki et al., 2005; Perrot and Rechler, 2005). Deacetylation of FOXO1 is mediated mainly by class II and III HDACs (Fig. 2) (Huang and Tindall, 2007). Upon exposure to stress stimuli, SIRT1 interacts with the LXXLL motif of FOXO1, resulting in retention in the nucleus and increased in DNA-binding activity (Fig. 2A) (Frescas et al., 2005; Nakae et al., 2006). Deacetylated FOXO1 elevates the expression of gluconeogenic genes (Fig. 2A) (Park et al., 2010). Furthermore, expression of gene encoding glucokinase (GK), a key enzyme in glucose utilization, is regulated through inter-relationship between SIRT1-deacetylated FOXO1 and hepatocyte nuclear factor 4alpha (HNF4α) (Fig. 2A) (Ganjam et al., 2009). Recruitment of HDAC3 (a class I of HDACs) by class IIa HDACs resulted in the deacetylation of FOXO1, which in turn upregulates the expression of gluconeogenic genes (Fig. 2A) (Mihaylova et al., 2011). Recently, SIRT2 has also been known to deacetylate FOXO1, resulting in decreased adipocyte size with improvement in whole-body insulin sensitivity (Fig. 2B) (Jing et al., 2007; Gross et al., 2009). In 3T3-L1 pre-adipocytes, the deacetylation of FOXO1 by SIRT2 acts as a critical modulator of FOXO1 activity, resulting in the transcriptional repression of the PPARγ promoter (Fig. 2B) (Jing et al., 2007). In addition, administration of adenoviral SIRT2 shRNA increases the acetylation of FOXO1, alleviating the suppression of PPARγ gene expression by inducing export of FOXO1 into the cytoplasm (Fig. 2B) (Jing et al., 2007; Wang and Tong, 2009).

**Table 2:** List of acetylated transcription factors that are involved in glucose and lipid metabolism

Transcription factor	Acetylase	Deacetylase	Amino acid	References
FOXO1	CBP/p300	SIRT1 SIRT2	K242, K245, K262	Matsuzaki et al. (2005), Jing et al. (2007)
CREB	CBP/p300	SIRT1	K91, K94, K136	Lu et al. (2003), Paz et al. (2014)
SREBP-1a	CBP/p300		K313, K324, K333	Giandomenico et al. (2003)
SREBP-1c	p300	SIRT1	K289, K309	Ponugoti et al. (2010)
SREBP-2	CBP/p300			Giandomenico et al. (2003)
FOXA2	p300	SIRT1	K259, K275	von Meyenn et al. (2013)
ChREBP	p300	SIRT1	K672	Bricambert et al. (2010), Marmier et al. (2015)
C/EBPβ	GCN5	HDAC1	K39, K98, K101, K102	Cesena et al. (2007), Wiper-Bergeron et al. (2007), Cesena et al. (2008)

### cAMP-responsive element-binding (CREB) protein

CREB is a transcription factor that binds to an 8-bp element known as the cAMP-response element (CRE) in the promoter regions of target genes (Altarejos and Montminy, 2011). CREB is expressed in several tissues that regulate the expression of genes related to neuronal differentiation, adipocyte differentiation, hepatic glucose levels, and lipid metabolism (Altarejos and Montminy, 2011; Ravnskjaer et al., 2013). Suppressing CREB using antisense-oligonucleotides (ASOs) prevents hepatic insulin resistance and steatosis-associated T2DM (Erion et al., 2009). Phosphorylation of CREB by glucagon is well characterized and enhances transcriptional activities (Fig. 2A) (Altarejos and Montminy, 2011). CBP is known to acetylate three lysine residues (Lys91, Lys94, and Lys136) located within the activation domain of CREB (Table 2) (Lu et al., 2003). Moreover, acetylation of CREB increases CREB-dependent transactivation (Fig. 2A) (Lu et al., 2003). Interestingly, the function of CREB in glucose and lipid metabolism has been suggested to be regulated by the crosstalk between acetylation and phosphorylation (Fig. 2A) (Paz et al., 2014).

### Sterol response element-binding proteins (SREBPs)

SREBPs are transcriptional regulators belonging to the basic helix-loop-helix leucine zipper (bHLH/LZ) family (Soyal et al., 2015). The SREBP family consists of SREBP-1a, -1c, and -2 (Shimano, 2009). SREBP-1c is primarily involved in fatty acid and TG biosynthesis, whereas SREBP-2 activates cholesterol biosynthesis (Soyal et al., 2015). In the feeding state, SREBP-1c stimulates both hepatic lipogenesis and glycolysis by upregulating target gene expression (Kim et al., 2004; Jeon and Osborne, 2012). One of the novel mechanisms regulating the transcriptional activities of SREBPs is PTMs, such as phosphorylation, ubiquitination, and acetylation (Fig. 2B) (Shao and Espenshade, 2012). In particular, acetylation has been shown to play a critical role in the regulation of SREBP transcriptional activity. SREBPs are acetylated by CBP/p300 through direct interactions (Giandomenico et al., 2003; Sundqvist and Ericsson, 2003). There are three potential acetylation sites in SREBP-1a (Lys313, Lys324, and Lys333) (Table 2) (Giandomenico et al., 2003). Among these, two lysine residues (Lys324 and Lys333) located at DNA-binding domain are important for transcriptional activation (Fig. 2B) (Giandomenico et al., 2003). Indeed, CBP/p300 plays a critical role in the regulation of SREBP-1a stability by interfering with the ubiquitination of these residues (Fig. 2B) (Giandomenico et al., 2003; Sundqvist and Ericsson, 2003). In contrast, SREBP-1a is deacetylated by SIRT1, resulting in a decrease in the stability and its association with hepatic lipogenic gene expression (Fig. 2B) (Walker et al., 2010). In parallel with SREBP-1a, acetylation and deacetylation of SREBP-1c occurs at Lys289 and Lys309 through the activity of p300 and SIRT1, respectively (Fig. 2B; Table 2) (Ponugoti et al., 2010). Notably, the acetylation of SREBP-1c is highly increased in DIO mice and depletion of hepatic SIRT1 in mice results in increased lipogenic gene expression (Ponugoti et al., 2010). In addition, overexpression of SIRT1 attenuates the transcriptional activity of SREBP-1c by decreasing stability and occupancy at the promoter of lipogenic genes (Fig. 2B) (Ponugoti et al., 2010). In a physiological state, reversible acetylation of SREBPs is dynamically regulated during fasting and feeding cycles (Walker et al., 2010). Function of acetylated SREBP-2 and its acetylation sites are not identified despite SREBP-2 is acetylated by CBP/p300 (Giandomenico et al., 2003). It may be possible that acetylation of SREBP-2 may have similar biological consequnences which observed in the acetylation of other subtypes SREBPs.

### Forkhead box A (FoxA) family proteins

FoxA family proteins, including FoxA1, -A2, and -A3 (also known as HNF3α, HNF3β, and HNF3γ) belong to a subfamily of the forkhead group containing a winged helix DNA-binding domain and play a crucial role in lipid metabolism (Lalmansingh et al., 2012). FOXA2 is a central regulator in hepatic lipid metabolism, with role in fatty acid oxidation, ketogenesis, and bile acid metabolism (Wolfrum et al., 2004). Interestingly, phosphorylation of FOXA2 by metabolic stress causes nuclear export with inhibition of its transcriptional activities (Fig. 2B) (Howell and Stoffel, 2009; Banerjee et al., 2010). Acetylation of FOXA2 occurs at multiple lysine residues (Lys6, Lys259, Lys264, Lys274, and Lys275) (Table 2) (van Gent et al., 2014). Among these, two residues (Lys259 and Lys275) are acetylated by p300 when glucagon is added (von Meyenn et al., 2013). In contrast to phosphorylation, acetylation of FOXA2 results in increased in their stability and transcriptional activity due to retention in the nucleus (Fig. 2B) (von Meyenn et al., 2013; van Gent et al., 2014). As expected, deacetylation of FOXA2 by SIRT1 has the opposite effects, causing reductions in stability and transactivation in the nucleus (Fig. 2B) (von Meyenn et al., 2013; van Gent et al., 2014). Overall, acetylation of FOXA2 may prevent the T2DM by upregulating the expression of β-oxidation and ketogenesis (Wolfrum et al., 2004; von Meyenn et al., 2013).

### Carbohydrate-response element-binding protein (ChREBP)

ChREBP is a bHLH/LZ transcription factor that functions in forming a heterodimeric complex with Max-like protein X (MLX) (Ma et al., 2006). ChREBP is a major mediator of the glucose effect independent of insulin action, which upregulates glycolysis- and lipogenesis-related genes, such as L-pyruvate kinase (L-PK), fatty acid synthase (FAS), acetyl-CoA carboxylase (ACC), and steroyl-CoA desaturase-1 (SCD-1) (Postic et al., 2007). The transcriptional activities of ChREBP are regulated by multiple PTMs, including phosphorylation, O-GlcNAcylation, and acetylation (Guinez et al., 2011; Filhoulaud et al., 2013). In the high-glucose state, ChREBP is acetylated by p300 at Lys672, which is located within the bHLH/LZ domain (Table 2) (Bricambert et al., 2010; Chen et al., 2010). In contrast to the other transcription factors mentioned above, acetylation of ChREBP does not affect nuclear-cytoplasmic shuttling (Fig. 2B) (Bricambert et al., 2010). Acetylated ChREBP readily binds to the ChREBP-responsive element (ChoRE) (Fig. 2B) (Bricambert et al., 2010). Recently, acetylation of ChREBP is increased by inhibiting the expression and activity of SIRT1 upon ethanol (EtOH) treatment (Fig. 2B) (Marmier et al., 2015). Consequently, hyper-acetylated ChREBP increases the expression of lipogenesis-related genes in the liver.

### CCAAT/enhancer-binding proteins (C/EBPs)

There are six members in C/EBP family (Nerlov, 2007). These proteins are bZIP transcription factors and are expressed in peripheral tissues (Nerlov, 2007). Among these, C/EBPβ plays an important role in hepatic glucose and lipid metabolism (Schrem et al., 2004). Modulation of transcriptional activities by C/EBPβ phosphorylation has been demonstrated (Park et al., 2004). Acetylation of C/EBPβ may also serve as a molecular switch for the regulation of transcription activities (Cesena et al., 2007; Wiper-Bergeron et al., 2007; Cesena et al., 2008). Acetylation of C/EBPβ at Lys39, which is located within the activation domain (AD), results in the transactivation of gene expression associated with adipogenesis (Fig. 2B; Table 2) (Cesena et al., 2007; Cesena et al., 2008). In glucocorticoid-stimulated pre-adipocyte differentiation, GCN5 is known to acetylate lysine residues (Lys98, Lys101, and Lys102) (Fig. 2B; Table 2) (Wiper-Bergeron et al., 2007). Acetylation of C/EBPβ increases the transcription of C/EBPβ target genes, such as C/EBPα and PPARγ by disrupting the interaction between C/EBPβ and the HDAC1 corepressor complex (Fig. 2B) (Abdou et al., 2011). At present, deacetylation of C/EBPβ and its biological significance are not clear. It is possible that deacetylation of C/EBPβ may have opposing effects to acetylation, similar to the phenomena observed for other transcription factors.

## PERSPECTIVES AND CONCLUSION

The epidemic of metabolic syndrome and T2DM has become a serious health problem in modern society. In the coming decades, these disorders will cause a much larger global problem, requiring physical, social, and economic expenses. In this review, we have summarized recent advancements in the understanding of gene regulation in the context of reversible acetylation/deacetylation of transcription factors and their roles in the regulation of gene involved in metabolic homeostasis, which are associated with the pathogenesis of metabolic syndrome. It has become clear that single or multiple acetylation residue(s) in transcription factors can trigger significant effects on whole-body energy homeostasis.

Exploring the key features of non-histone protein acetylation, including that of transcription factors will be critical for understanding fine control of whole body metabolism. Thus understanding the molecular mechanisms and physiological relevance of acetylation is of great interest, considering the therapeutic potential of these processes in the development of drugs combating metabolic syndrome and T2DM.
